# Case Report: A sailor's knot in the heart: percutaneous retrieval of a knotted temporary pacing lead

**DOI:** 10.3389/fcvm.2026.1756548

**Published:** 2026-02-02

**Authors:** Roberto Scacciavillani, Simone Filomia, Gaetano Pinnacchio, Gianluigi Bencardino, Tommaso Sanna, Gemma Pelargonio

**Affiliations:** 1 Department of Cardiovascular Sciences, Fondazione Policlinico Universitario A. Gemelli IRCCS, Rome, Italy; 2 Department of Cardiovascular and Thoracic Sciences, Catholic University of the Sacred Heart, Rome, Italy

**Keywords:** atrioventicular block, electrophysiology, knot, lead extraction methods, temporary pacemaker complication

## Abstract

**Background:**

Temporary transvenous pacing in patients with permanent atrial fibrillation and significant tricuspid valve disease is technically challenging, especially in the absence of fluoroscopic guidance. Catheter looping and knot formation are rare but potentially hazardous complications.

**Case summary:**

An 82-year-old woman with permanent atrial fibrillation and severe mitral and tricuspid valve regurgitation presented with complete atrioventricular block and a ventricular escape rhythm at 28 bpm. A temporary transvenous pacing catheter inserted via the right internal jugular vein failed to achieve consistent ventricular capture and became entrapped at 45 cm, forming a knot near the venous introducer. Given the patient's frailty and high surgical risk, a multidisciplinary team opted for a fully percutaneous strategy for removal. After definitive pacemaker implantation, the knotted catheter was successfully retrieved using a stepwise approach: engagement of the loop with a deflectable ablation catheter, externalization through a femoral sheath, and extraction using a 13-F dilator sheath to minimize venous trauma.

**Discussion:**

This case highlights procedural pitfalls during emergency temporary pacing without fluoroscopy and illustrates a safe, creative percutaneous solution for knot retrieval using tools familiar to electrophysiology and lead extraction operators.

## Introduction

Temporary transvenous pacing is an essential intervention for hemodynamically significant bradyarrhythmias; however, its execution can be particularly challenging in patients with permanent atrial fibrillation and severe tricuspid valve pathology. In this population, the absence of atrial capture, distorted right-sided anatomy, and limited catheter support increase the likelihood of unsuccessful ventricular pacing. When fluoroscopy is unavailable - such as in emergency or resource-limited settings - catheter manipulation becomes even more difficult, predisposing to excessive advancement, venous looping, and, in rare cases, knot formation.

Knot entrapment is an uncommon but well-recognized complication, most frequently described with Swan–Ganz catheters, yet it may also occur with temporary pacing leads and can necessitate complex percutaneous retrieval techniques or even surgical extraction (1, 2).

We report a case of pacing-catheter knotting at the brachiocephalic–superior vena cava junction, successfully managed through a fully percutaneous approach. This case highlights the mechanical challenges inherent to blind right-sided catheterization and underscores the value of repurposing electrophysiology tools to facilitate safe problem-solving in urgent clinical scenarios.

## Case presentation

**Figure 1:**
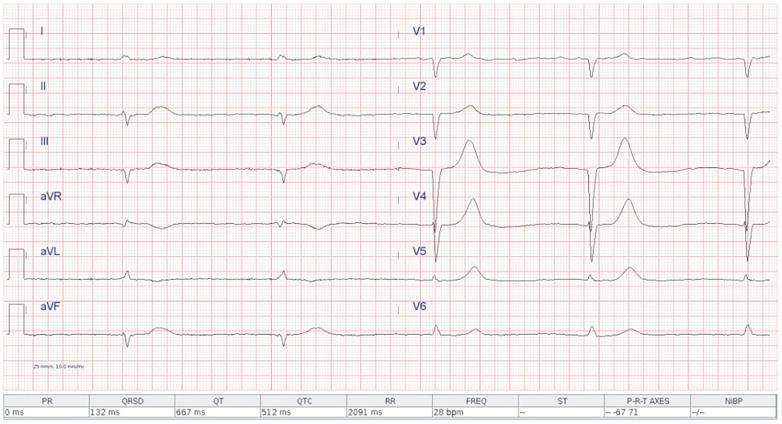
Admission ECG showing atrial fibrillation with complete atrioventricular block and a ventricular escape rhythm at 28 bpm.

An 82-year-old woman with known permanent atrial fibrillation presented to the emergency department overnight with asthenia, nausea, and bradycardia. The initial ECG showed atrial fibrillation with complete atrioventricular block and a ventricular escape rhythm at 28 bpm (Figure 1). She was stable from a respiratory standpoint, alert and cooperative, with signs of systemic congestion (jugular venous distension and peripheral edema). She was admitted to the cardiac intensive care unit, where an isoproterenol infusion was initiated at 0.03 µg/kg/min and progressively uptitrated. Owing to persistent rhythm instability despite isoproterenol, a temporary transvenous pacemaker was planned, with definitive pacemaker implantation scheduled for the following morning.

Transthoracic echocardiography showed preserved biventricular systolic function; biatrial enlargement; a sclerocalcific mitral valve with severe mixed stenosis–regurgitation; severe functional tricuspid regurgitation; a dilated inferior vena cava; and an estimated pulmonary artery systolic pressure of 45 mmHg.

**Figure 2:**
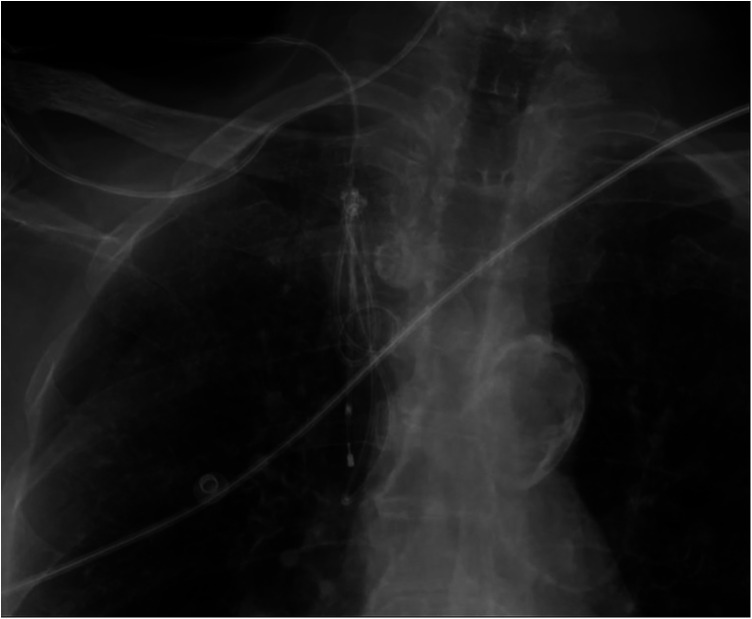
Chest x-ray detail showing the pacing lead loops within the brachiocephalic trunk and superior vena cava, with the knot trapped distal to the introducer and the catheter tip located in the superior vena cava.

A 7-Fr, 11-cm venous introducer was placed in the right internal jugular vein. In the absence of bedside fluoroscopy, the procedure was performed under ultrasound guidance with a second operator, and a 5-Fr, 110-cm balloon-tipped temporary pacing catheter (Spike Flow Bipolar Pacing Catheter, FIAB) was advanced. Despite correct connection to the generator and maximal output (20 mA) in VOO mode, there was no atrial capture (as expected in permanent atrial fibrillation) and no consistent ventricular capture, apart from sporadic beats when the catheter approached the tricuspid annulus. Ultrasound guidance was limited by a poor subcostal window and a suboptimal apical view in the supine position. After multiple maneuvers - withdrawal with the balloon deflated and readvancement with the balloon inflated - the catheter was advanced up to 55 cm without achieving capture. During subsequent withdrawal, the catheter became stuck at approximately 45 cm. A chest radiograph revealed a double loop of the pacing catheter with entrapment of a knot near the venous introducer (Figure 2). Meanwhile, the ventricular rate improved to 50–55 bpm on high-dose isoproterenol, so further invasive maneuvers were deferred overnight.

The following morning, after a multidisciplinary discussion involving the electrophysiology, interventional cardiology, and vascular surgery teams, the plan was to proceed first with implantation of a single-chamber permanent pacemaker, followed by a percutaneous attempt to untie the knot and remove the temporary pacing catheter.

After ultrasound-guided axillary vein puncture, a single-chamber pacemaker was implanted to ensure a stable paced rhythm during subsequent manoeuvres.

Two retrieval options were considered: tightening the knot as much as possible and attempting extraction through a larger femoral sheath, or surgical venotomy with direct retrieval. Because the patient was elderly, frail, and anticoagulated—and given the high perceived surgical risk—the team elected to attempt a percutaneous approach first, reserving surgery as a bailout option.

A right femoral vein approach was obtained using a 12-F introducer. The proximal segment of the temporary pacing catheter (the portion exiting the internal jugular vein) was cut at approximately 50 cm - the point where it had become fixed and could no longer be withdrawn**.**

After a first attempt with a decapolar catheter (Boston Scientific Dynamic deflectable catheter), a deflectable ablation catheter (Celsius D curve, Biosense Webster) was used to engage the loop, apply adequate torque, and draw the knotted catheter into the inferior vena cava (see Supplementary Video S1).

**Figure 3:**
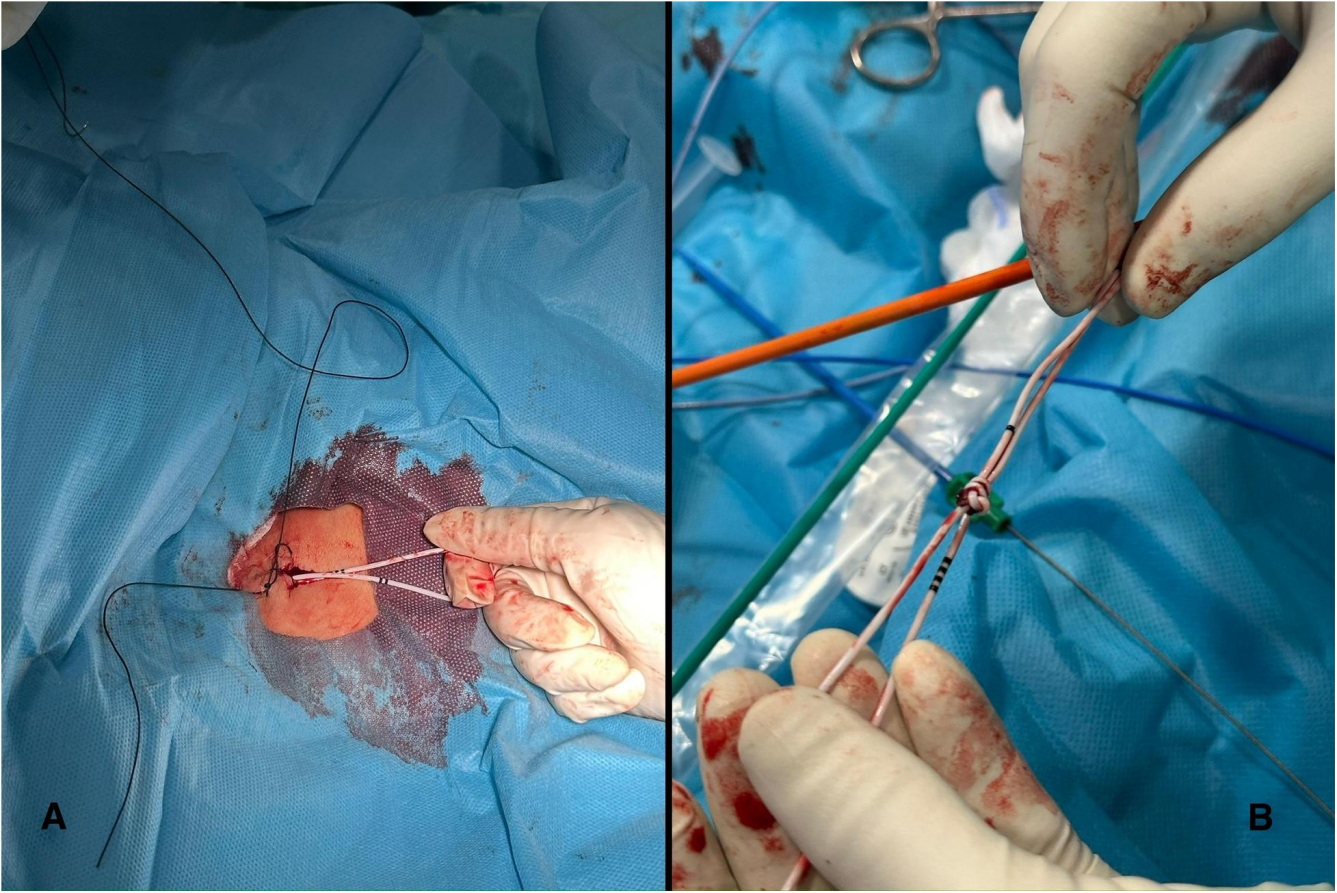
**(A)** (left): the externalized loop outside the femoral vein after removal of the 12-F sheath. (B) (Right): the knot after successful extraction. Note how the four black markers on the catheter correspond to 40 cm in length.

The loop was then grasped with a bioptome (7F Cordis), advanced into the introducer and exteriorised. However, because the irregularly shaped knot did not pass smoothly through the introducer (see Supplementary Video S1), the 12-F sheath was removed, leaving the externalised loop outside the femoral vein (see Figure 3A).

Drawing on experience from percutaneous lead extraction, a 13-F mechanical dilator sheath with a radiopaque band running on the side from the tip to the body (Cook Medical) was advanced to encompass the loop of the temporary pacemaker and allow extraction. The dilator was advanced coaxially toward the knotted portion of the temporary pacing lead. The sheath was deliberately positioned to capture the loop of the temporary pacemaker, creating a stable channel through which the knot could be gradually retrieved. This technique enabled controlled application of traction while protecting the venous wall and adjacent soft tissues, thereby allowing removal of the knotted lead with minimal tissue injury and a lower risk of venous dissection (see Supplementary Video S1 and Figure 3B). Hemostasis was achieved using a figure-of-eight suture and a Safeguard compression device, avoiding the need for both surgical venotomy and surgical closure.

## Discussion

Temporary transvenous pacing in patients with permanent atrial fibrillation and significant tricuspid valve disease is inherently challenging, particularly when fluoroscopic guidance is unavailable. In such settings, catheter manipulation relies almost exclusively on tactile feedback and limited ultrasound visualization, which are often insufficient to ensure accurate positioning within the right atrium and ventricle. As illustrated by this case, repeated advancement of the pacing catheter in the absence of reliable ventricular capture increases the risk of redundancy within the venous system, predisposing to excessive looping and, ultimately, knot formation.

Looping and knotting of temporary pacing catheters are rare but well-recognized complications, associated with significant risks including catheter entrapment, venous injury, and, in some cases, the need for surgical extraction. Early recognition is essential: radiographic evidence of an abnormal catheter course should prompt immediate cessation of further manipulation, reassessment of the strategy, and consideration of alternative tools or access routes.

In previously reported cases, where the knot was smaller and no major loop had formed, percutaneous retrieval via a femoral approach using a snare has proven effective (3, 4). Other techniques for unknotting have also been described, such as advancing a long sheath along the same venous route and “capturing” the knot within the sheath to facilitate removal (5). However, in our case this strategy would have required introducing a large-bore sheath through the internal jugular vein, which was deemed undesirable given the patient's frailty and the increased risk of vascular trauma.

Our case is notable for several technical aspects. First, a steerable ablation catheter with an acute D-curve was employed to engage and mobilize the knotted loop - an approach that proved more effective than a conventional snare in this anatomical context, as the loop offered a more stable and accessible target than the floppy catheter tip. Second, although the loop could be externalized through the femoral introducer, the knot itself remained too bulky and irregular to traverse a 12-F sheath safely. Direct traction through the soft tissues without an introducer would have entailed a substantial risk of venous or subcutaneous injury. To minimize trauma, a 13-F mechanical dilator sheath was advanced to gently dissect the perivascular tract and allow controlled, atraumatic extraction of the knot and pacing lead.

Tools designed for the capture of pacing leads include the needle eye snare, the goose-neck, the one-snare and the ensnare. However, we opted for this alternative strategy in this case given the presence of a big knot with a large loop, since we perceived that the ablation catheter allowed us to hook the loop and apply more torque force.

Another option would have been to implant a leadless pacemaker and then use the sheath to remove the knotted temporary pacing lead. However, leadless pacemakers are not readily available at our Institution, and we have to request the material to the manufacturer for every single case. Considering the urgency of the situation we did not consider this alternative.

This case underscores the importance of anticipating mechanical complications during temporary pacing in anatomically challenging patients and highlights how tools commonly used in electrophysiology and percutaneous lead extraction can be repurposed to enable safe, minimally invasive retrieval. Multidisciplinary collaboration was instrumental in choosing a percutaneous strategy over high-risk surgical venotomy, ultimately resulting in successful extraction with no vascular complications.

## Data Availability

The raw data supporting the conclusions of this article will be made available by the authors, without undue reservation.

## Supplementary material

Supplementary Video S1 Knot retrieval (See case presentation text for details).
